# MYT1L in the making: emerging insights on functions of a neurodevelopmental disorder gene

**DOI:** 10.1038/s41398-022-02058-x

**Published:** 2022-07-22

**Authors:** Jiayang Chen, Allen Yen, Colin P. Florian, Joseph D. Dougherty

**Affiliations:** 1 Department of Genetics, Washington University School of Medicine, 660 S. Euclid Ave, Saint Louis, MO 63108 USA; 2 Department of Psychiatry, Washington University School of Medicine, 660 S. Euclid Ave, Saint Louis, MO 63108 USA; 3 Intellectual and Developmental Disabilities Research Center, Washington University School of Medicine, 660 S. Euclid Ave, Saint Louis, MO 63108 USA

**Keywords:** Molecular neuroscience, Autism spectrum disorders

## Abstract

Large scale human genetic studies have shown that loss of function (LoF) mutations in MYT1L are implicated in neurodevelopmental disorders (NDDs). Here, we provide an overview of the growing number of published MYT1L patient cases, and summarize prior studies in cells, zebrafish, and mice, both to understand MYT1L’s molecular and cellular role during brain development and consider how its dysfunction can lead to NDDs. We integrate the conclusions from these studies and highlight conflicting findings to reassess the current model of the role of MYT1L as a transcriptional activator and/or repressor based on the biological context. Finally, we highlight additional functional studies that are needed to understand the molecular mechanisms underlying pathophysiology and propose key questions to guide future preclinical studies.

## Introduction

Human genetic studies recently associated the gene Myelin Transcription Factor 1 Like (*MYT1L)* with neurodevelopmental disorders (NDDs) [1–9]. Specifically, MYT1L loss of function (LoF) is associated with intellectual disability (ID) and autism spectrum disorder (ASD), while MYT1L duplication has been observed in patients with schizophrenia (SCZ) [10]. Yet, the mechanism by which MYT1L variants contribute to disease pathology is still unknown.

MYT1L, along with Myelin Transcription Factor 1 (MYT1) and Suppression of Tumorigenicity 18 (ST18/MYT3), is part of the three-gene MYT/neural zinc finger (NZF) transcription factor (TF) family. These TFs are characterized by DNA binding C2HC-type zinc fingers, and a MYT1 domain, which is hypothesized to function as a transcriptional repressor [11, 12]. While all three TFs are found to be expressed in the developing brain, MYT1L has specifically been shown to enhance neuronal differentiation [11,13]. Seminal studies have shown that overexpression of ASCL1 and BRN2 reprograms fibroblasts into functional neurons in vitro and the addition of MYT1L significantly increases conversion efficiency [14]. However, the exact role of MYT1L during this transdifferentiation process remains poorly understood. As a member of MYT/NZF protein family, it is thought that MYT1L represses its target genes’ expression, reminiscent of the known repressive functions of MYT1. Indeed, in vitro neuronal transdifferentiation studies demonstrated that MYT1L represses non-neuronal gene expression, while promoting neuronal differentiation [11]. On the other hand, both in vitro and in vivo studies indicate MYT1L can activate gene expression with a comparable magnitude to reported repression, suggesting that it can also function as an activator [15, 16]. Further studies are needed to resolve its true molecular function in biologically relevant contexts.

In this review, we first discuss the association between MYT1L variants and human disease phenotypes. Then, we integrate results from in vitro studies to summarize the known cellular and molecular functions of MYT1L. Finally, we identify key outstanding questions and propose future directions for MYT1L studies with a focus on cutting-edge techniques that could elucidate MYT1L’s function in even greater depth. We hope this review will serve as a primer on the current state of research into this emerging NDD-associated gene and highlight opportunities for future investigation.

## The association of MYT1L mutation and human disease

Human genetic studies have identified genetic mutations in transcription factors and chromatin remodelers (*MECP2*, *CHD8*, *SETD5*, etc.) as causes for various forms of neuropsychiatric disorders, ID, ASD, and SCZ [2, 3, 7, 17–19]. One of these newly associated factors is MYT1L.

**Fig. 1:**
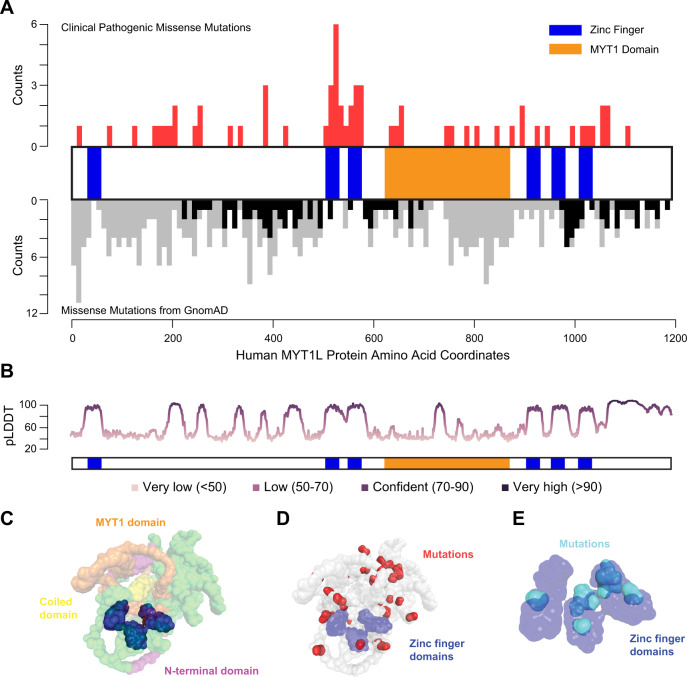
Schematic of human MYT1L domains and predicted protein structure by AlphaFold. **A** Distribution of missense mutations described in clinical studies (top, red) compared to a general population sample (gnomAD, bottom, with gray bars displaying all missense mutations and black bars displaying ‘possible damaging mutations’ as predicted by PolyPhen2). ‘Possible damaging mutations’ in the general population are largely excluded from the regions mutated in clinical samples. **B** AlphaFold’s calculated confidence measure (pLDDT score) per-residue of the model’s prediction based on the IDDT-Cα metric. **C** 3D AlphaFold structure (AF-Q9UL68-F1) prediction of MYT1L protein showing the N-terminal domain (magenta), MYT1 domain (orange), coiled domain (yellow), and six zinc finger domains (blue) coming in proximity with each other to form a putative DNA-binding pocket. Unannotated regions are shown in green. (https://alphafold.ebi.ac.uk/entry/Q9UL68). **D** Loss of function mutations from patient reports are found throughout the protein. Those not within the annotated zinc finger domains (blue) are shown in red. **E** Isolated and magnified view of the zinc finger domains (blue) shows patient mutations (cyan) cluster in the zinc fingers.

With the increased integration of genome sequencing into the clinic over the last 10 years, *MYT1L* mutations, mostly de novo, have consistently been found in patients with early onset neurological disorders. Currently, there are over 100 described patients with MYT1L mutations, with 80% of them harboring potential *MYT1L* LoF mutations and others harboring *MYT1L* partial duplications [5, 8, 10, 20]. *MYT1L* LoF mutations include deletions, frameshift, and single nucleotide variations (SNVs), which are predicted to cause decreases in mRNA production or aberrant protein functions. Notably, missense mutations from clinical but not general-population studies cluster in the central zinc finger domains and the MYT1 domain [21, 22] (Fig. 1A), the most confident structures predicted by AlphaFold (Fig. 1B, Supplemental Videos 1, 2), indicating these domains might be crucial for the protein’s functions [23, 24]. Among patients with *MYT1L* LoF mutations, ID, ASD, and developmental delay are the most common symptoms. Other phenotypes include seizures, syndromic obesity, microcephaly, macrocephaly, and muscular hypotonia. This constellation of symptoms has now been recognized as MYT1L Syndrome or 2p25.3 Deletion Syndrome [5, 8, 10, 20]. In addition, most patients with *MYT1L* partial duplications were reported to either have ID, ASD, or both. It seems these developmental impacts of MYT1L haploinsufficiency indicate a well-conserved role for the protein: across two labs, with independently generated lines, MYT1L haploinsufficient mice were also shown to have obesity, hyperactivity, and social deficits [15, 25].

Finally, regarding *MYT1L* duplications in humans, although 33% of *MYT1L* duplication patients presented with SCZ exclusively, all but one of those duplications contain neighboring gene *PXDN*, indicating *MYT1L* may not be the only contributing factor in the region for SCZ risk [10]. The association of both LoF and putative duplications with disease indicates that neurobiology is very sensitive to the levels of MYT1L activity and identifying the loci that are influenced by altered MYT1L levels might aid in understanding the downstream pathophysiology. Therefore, in the following sections, we summarize previous studies on MYT1L to provide mechanistic insights into its cellular and molecular functions under different contexts.

## Cellular functions of MYT1L

### MYT1L functions to promote neuronal maturation

Neuronal identity is determined by the effects of a combination of basic helix-loop-helix (bHLH) TFs (i.e., ASCL1, NEUROD1, and NEUROG1) as well as other developmentally expressed TFs such as BRN2 and MYT1L. In vitro overexpression studies have shown that the pioneer factor ASCL1 is sufficient for induction of neuronal traits, but overexpression in combination with other factors such as BRN2, and especially, MYT1L is necessary for efficient fibroblasts conversion to neurons as well as the maturation of the induced neurons (iNs) [11, 26, 27]. Ultimately, many of these studies suggest that MYT1L and other members of the MYT family primarily function to preserve neuronal phenotypes as it has been shown that MYT1L is mostly expressed during the post-specification phase when cell populations have become post-mitotic. Furthermore, none of the MYT family members were observed to be expressed by in situ hybridization in germinal zones containing mostly undifferentiated cells [13, 28], and very little overlap (5%) was seen with SOX2 positive progenitors [15]. Interrogation of specific domains of MYT1L has further defined its role in neuronal conversion. For example, Mall et al. [11] showed that, when fused to an activating element (VP64), the DNA binding domains of MYT1L displayed a dominant-negative effect on ASCL1-mediated neuronal conversion. In addition, just a 423-amino-acid fragment (i.e., amino acids 200–623), which contains the N-terminal domain and the middle two zinc fingers, was functionally indistinguishable from full-length MYT1L. Surprisingly, this fragment does not contain the MYT1 domain.

In contrast to the overexpression studies discussed above, knockdown of MYT1L via short hairpin (sh) RNAs resulted in a reduction of neuronal maturation gene programs such as neurite outgrowth, axonal development, synaptic transmission, and extracellular matrix composition, which hints that MYT1L also acts as an activator [29]. It has also been reported that MYT1L was found to be deleted (~5%) and downregulated (>80%) in glioblastomas, suggesting that gliomagenesis requires neutralization of terminal neural differentiation [30]. Furthermore, others have shown that MYT1L and MYT1 expression can slow tumor growth in glioblastoma cell line models via repression of pro-proliferative genes [31]. However, impacts on glia in vivo are likely not direct since MYT1L expression has not been consistently observed in glia [15, 32].

**Fig. 2:**
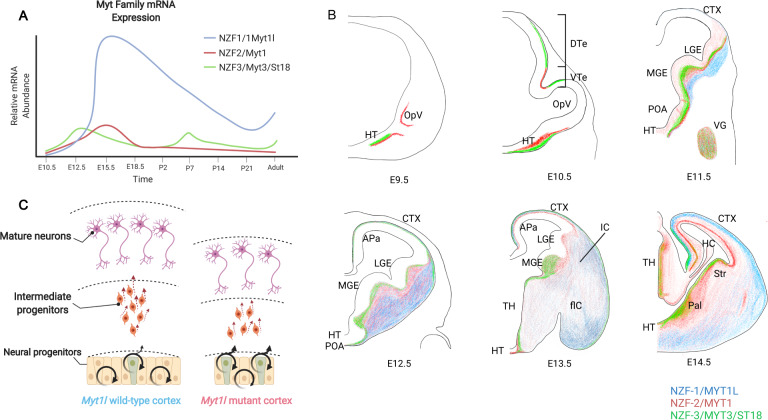
Mouse embryonic brain expression patterns of MYT family transcription factors. **A** Quantitative RT-PCR summarized as relative mRNA expression of Myt1 (red), Myt1l (blue), and Myt3 (green) in the developing mouse from E10.5 to adult, adapted from [13]. **B** Color coded summary of published in situ hybridization data from Matsushita et al. [13] showing the spatial expression pattern of MYT1, MYT1L, and MYT3 in the developing cortex. **C** The diagram shows a hypothesized mechanism of microcephaly in Myt1l mutant mice at E14. APa, archipallium; BG, basal ganglia; CTX, cortex; DTe, dorsal telencephalon; fIC, fibers of the internal capsule; HC, hippocampus; HT, hypothalamus; IC, internal capsule; LGE, lateral ganglionic eminence; MGE, medial ganglionic eminence; OpV, optic vesicle; Pal, pallidum; POA, preoptic area; Str, striatum; TH, thalamus; Vg, trigeminal ganglion; VTe, ventral telencephalon.

Spatiotemporal expression of MYT family TFs is finely tuned across development, specifically during neuronal maturation. Of the MYT family, Myt1 and Myt3 are expressed the earliest at embryonic (E) day 9.5 as suggested by in situ hybridization [13]. Quantitative RT-PCR results showed that Myt1 and Myt1l were upregulated from E10.5 to E15.5 and then downregulated postnatally (Fig. 2A) [13]. In addition, Myt1l mRNA levels increase across neurogenesis in mice, and low levels are sustained in adulthood, which mirrors human expression patterns [13]. In mice, MYT1L protein levels were sustained from E14 (beginning) to postnatal (P) day 1 and declined thereafter [15], but remained detectable indefinitely. The earliest time point of detectable Myt1l expression occurs at E9.5 in the ventrolateral portion of the spinal cord, again where newborn neurons are found. In addition, at E12.5, BrdU staining to identify proliferating cells hardly overlapped with Myt1l expression, further supporting that Myt1l-positive cells were mostly post-mitotic [13]. Indeed, across the multiple CNS regions examined (spinal cord, hindbrain, midbrain, cortex, and retina), Myt1l mRNA was upregulated when neurons began to differentiate (Fig. 2B) and overlapped with markers of neurons. Overall, analysis of Myt1l expression pattern and time course further supports the assumption that it is responsible for neuronal maturation and preservation of cell fate.

Several in vivo studies have also shed light on MYT1L’s necessity for neuronal maturation. In zebrafish, knocking down human MYT1L orthologs, myt1la and myt1lb, by antisense morpholinos (MO) results in almost complete loss of oxytocin (OXT) and arginine vasopressin (AVP) in the neuroendocrine pre-optic area of the hypothalamus, suggesting MYT1L LoF might affect neuroendocrine system development [5]. This could either represent loss of these neurons, or loss of their maturation since neuropeptide expression occurs relatively late in neuronal maturation [33]. In a MYT1L Syndrome mouse model that displays MYT1L haploinsufficiency, precocious neuronal differentiation from progenitors to immature neurons was observed upon MYT1L loss during early brain development [15] (Fig. 2C). This suggests MYT1L LoF leads to loss of proliferating cells during development and correspondingly a smaller brain in the adult, providing a mechanistic explanation for the human patients’ microcephaly. In addition, assessment in adults revealed MYT1L heterozygous mice show impaired neuronal maturation in terms of transcriptional profiles, neuronal morphology, and potentially neuronal electrical properties [15]. In summary, MYT1L may have multiple roles in neurodevelopment, with strong evidence that at least one may be promoting neuronal maturation.

## Molecular functions of MYT1L

### Functional domains within MYT1L

Structurally, MYT family TFs have several domains that may define their functions and many initial studies of MYT family TFs focused on the characterization of structural domains and DNA binding [32, 34]. Amino acid sequence analysis of MYT1L isolated from rat pituitary and cerebellum cell lines showed that the protein has six zinc finger domains (Cys-Cys, His-Cys) that are organized into clusters with one at the N-terminus, a pair upstream of the MYT1 domain, and three at the C-terminal domain [34]. Using AlphaFold [23, 24] to model the structure of MYT1L, these six zinc finger domains are predicted to come together to form a DNA binding pocket (Fig. 1C, Supplemental Video 2). *MYT1L* LoF mutations based on patient reports can be found throughout all domains of the protein (Fig. 1D) with a notable cluster of mutations within the central zinc finger domains (Fig. 1E). The N-terminus contains a highly acidic region, and a Ser/Thr-rich region between the two clusters of zinc fingers [11].

The zinc fingers are thought to be involved in DNA-binding. The core sequence recognized by the MYT1L zinc fingers, specifically from the two zinc fingers upstream of the MYT1 domain, is GAAAGTT [34]. An additional GTT that resides 4 bp 5′ of the core sequence element was observed in the DNA binding motif when testing the construct with the three zinc fingers at the C-terminal end of the protein. Competitive binding assays showed that AAGTT was the most crucial for binding [34], which is consistent with the motif reported from ChIP-seq experiments [11]. Further, two additional purines at the 5′ end (RRAAGTT) are preferred for optimal binding. The N-terminus, which is the least conserved domain among the MYT family of TFs, with its highly acidic region that is enriched for Glu/Asp residues, has been implicated in transcriptional activation [16, 34], a function that appears dispensable for production of transdifferentiated neurons [11].

The N-terminus, MYT1 domain, and C-terminus of MYT1L also contain structural components that are unique to the MYT family. The MYT1 and C-terminal domains are highly conserved across the MYT family, and the MYT1 domain contains a Ser/Thr-rich region in both MYT1 and MYT1L [11, 34]. Several studies used these specific fragments of MYT1 and MYT1L to characterize the transcriptional effects of each protein, which is discussed more in the final section of the review.

## Key questions and future directions

### What are the targets of MYT1L?

The emerging role of MYT1L in NDDs highlights the importance of a more complete understanding of MYT1L within transcriptional networks, and the identification of its targets is essential to systematically study the downstream consequences of MYT1L haploinsufficiency. Thus far, identification of MYT1L-regulated genes has been addressed via in vitro overexpression or knockdown studies followed by analysis of changes in gene expression. For example, fibroblast gene signatures were found to be downregulated by RNA-seq upon induction of iNs [11]. In particular, MYT1L repressed Notch signaling and Hes1 to promote neurogenesis, and acute shRNA knockdown of MYT1L phenocopied Notch gain-of-function.

While studies of gene expression are informative, they do not distinguish between direct and indirect regulation. Direct targets have been identified by ChIP-seq primarily in fibroblasts overexpressing MYT1L and with limited samples from endogenous MYT1L in the developing mouse brain [11]. Within a subset of ChIP-seq peaks, the previously described AAGTT motif was enriched within downregulated genes in fibroblasts, consistent with the hypothesis of MYT1L’s role as a transcriptional repressor. However, the direct targets in vivo tended to show a loss of chromatin accessibility and expression in MYT1L mutants, suggesting the loss of an activator [15]. It may also be that MYT1L binding has distinct functions at different sites or occurs in a context-dependent manner. Regardless, little is known about MYT1L binding at other time points (embryonic vs. adult), or how binding and activity changes in haploinsufficiency. To detect subtle effects and to understand the observed changes in chromatin and gene expression in MYT1L models, an atlas of MYT1L binding sites is needed (e.g., a high-quality time course ChIP-seq or CUT&RUN experiment). In addition, binding peaks can be quantified to determine changes in TF binding in a concentration-dependent manner, which will be critical in identifying differentially bound genomic regions and gene expression in MYT1L haploinsufficient mice.

How the impact of MYT1L loss changes across cell types is also unclear. There have been significant developments in multiomic assays for directly linking chromatin accessibility to the transcriptome within the same single cells. Coupled with the MYT1L syndrome mouse model, this would be a powerful assay to address the questions of how MYT1L loss affects chromatin state and whether this loss disproportionately affects a specific neural cell subtype. One inherent limitation to most “omic” methodologies is that they only capture a snapshot of the “-ome” at the time of sample collection. An emerging method, Calling Cards, seeks to preserve historical cell states by recording TF-DNA interactions over time [35, 36]. This cumulative history of the location of TF binding across time can generate a unique dataset to link TF binding, transcription, cell fate decisions, and developmental trajectories in single cells, which might be of interest to apply here. Overall, a more complete catalog of MYT1L targets in specific neural subtypes will be key to understanding the mechanisms underlying the observed phenotypes following LoF mutation.

### What are the modes of MYT1L TF binding?

Our ability to associate TF binding with gene expression is still imperfect, and it is unclear if MYT1L binds in a direct mode to DNA, or if it requires cofactors (indirect mode) for some DNA interactions. ChIP-seq and motif discovery algorithms are efficient at identifying direct TF binding sites (usually within ~50 bp). However, these methods also capture indirect TF-DNA interactions through other TFs that bind cooperatively. This becomes apparent when sequences bound in vivo differ from those that are found in vitro [37]. Also, as patients are MYT1L haploinsufficient, one could speculate that MYT1L binding targets are dependent on the concentration of MYT1L. In addition to motif sequence specificity and intra- and intermolecular TF interactions, adjacent flanking sequences are also key determinants of TF binding and regulation. Thus, additional studies and methodologies that can differentiate between direct and indirect TF binding and link these dynamics to cis regulatory elements will be important to understanding the consequences of altered MYT1L levels on gene expression.

Based on ChIP-seq data of reprogrammed iNs, MYT1L binding sites are enriched in transcription start sites, whose corresponding genes were often repressed during fibroblast-to-neuron transdifferentiations [11]. However, such overexpression of MYT1L could lead to interactions not found under physiological conditions. Moreover, the accessibility of putative MYT1L targets may be influenced by the indirect effects of reprogramming fibroblasts into neurons. MYT1L ChIP-seq provides a readout of direct targets of MYT1L, but a complete understanding of its DNA binding motif is often difficult or impossible to glean from this data alone. Indeed, most peaks do not show the known MYT1L motif, suggesting that much of MYT1L binding is indirect. Thus, MYT1L ChIP-seq is important in determining TF binding in a specific biological context, but complementary methods are still needed to understand binding preferences. SELEX (also known as SAAB or CASTing), is a cell-free assay that has been adapted to screen for sequences that bind a TF of interest [38, 39]. Both high and intermediate affinity sequences can be captured and used to characterize binding affinities. This method enabled the de novo motif discovery and confirmation of MYT1L binding sites [11], but it would be difficult to assay flanking sequences and intermolecular interactions that can affect MYT1L binding in vivo. Several open questions remain. Does MYT1L require cooperative binding, or can it function independently? Although MYT1L is not known to have pioneering abilities, can its binding prevent chromatin remodeling/nucleosome repositioning? Can cofactors alter the motif sequence specificity of MYT1L? A better understanding of MYT1L TF biology and the determinants contributing to its binding will be critical in understanding its functions and the consequences of its dysfunctions.

### Is MYT1L an activator or a repressor?

Once MYT1L binds to DNA, whether it functions as a transcriptional activator, repressor, or both, is still not clearly understood. In vitro transdifferentiation studies have represented MYT1L as a repressor of non-neuronal gene programs [11, 14], while other in vivo studies have found evidence that MYT1L activates neuronal genes [15, 29]. Early in vitro studies show that MYT1L was able to activate a hRARβ promoter-luciferase reporter as well as a Pit-1 enhancer/promoter luciferase reporter in CV-1 and HeLa cells [34]. Furthermore, MYT1 and MYT1L were directly compared using an in vitro reporter with a synthetic promoter carrying seven copies of the AAAGTTT motif separated by nine nucleotides [16]. In this assay, overexpression of full-length MYT1 repressed transcription while overexpression of full-length MYT1L activated transcription of the reporter in HeLa, A549, and U87 cells, which all have relatively low or no endogenous MYT1 and/or MYT1L expression. In cultured neuronal cells, shRNA-mediated knockdown of MYT1L resulted in reduced expression of neuronal transcripts associated with neurite outgrowth, axonal development, and synaptic transmission [29]. This is consistent with recent data from a germline MYT1L heterozygous mouse model showing increased expression of “early fetal” genes in prefrontal cortex of adult mice, resulting in an immature transcriptional signature compared to wild-type (WT) mice [15].

MYT1L also contains a repressive MYT1 domain. Compared to the N-terminal activation domain, the MYT1 domain appears to be highest conserved region second to the middle and C-terminal zinc fingers, containing the Ser/Thr-rich region in MYT1 and MYT1L [11, 34] (Fig. 1A), and appears repressive in most studies so far. Mechanistically, through a yeast-two-hybrid screen, the central domain of MYT1 was shown to interact with the corepressor SIN3B. Since this region is conserved across the MYT family, it was also shown that MYT1L interacted with SIN3B using a Gal4 assay [12], and other studies have supported the conclusion that the central, MYT1 domain can interact with the corepressor SIN3B [11]. Specifically, the interaction between MYT1 and MYT1L with SIN3B can result in transcriptional repression via histone deacetylase (HDAC) interaction with SIN3B [12]. When directed to promoter regions by MYT1 and MYT1L, the SIN3B-HDAC complex can remove activating chromatin modifications, resulting in less accessibility and ultimately, repression [12].

The seemingly divergent functions of the activating N-terminal domain and repressive MYT1 domain make it challenging to classify MYT1L as a transcriptional activator or repressor. Altogether, these focused studies on the molecular domains of MYT1L suggest that the role of MYT1L is context dependent and may largely function as an activator in vivo. Follow-up studies are needed to determine if the role of MYT1L remains the same in adulthood after neurodevelopment has been completed.

To analyze the molecular and cellular role of MYT1L during neurodevelopment, a detailed time-course analysis of chromatin accessibility and TF binding is required. Single-cell/nuclei technologies can be leveraged to identify the cis regulatory landscapes and trajectories of the different cell types that make up the brain [40]. This general approach can be used with the MYT1L Syndrome mouse model to map altered gene regulatory programs and resulting impact on cellular proportions upon loss of MYT1L. Traditional methods to assay the TF activation or repression utilize fluorescence or luciferase-based reporter constructs for a quantitative readout of downstream activities. While these are highly sensitive and reproducible, they are not suitable for high-throughput screening of hundreds of putative regulatory elements. Massively parallel reporter assays (MPRAs) are an approach that can be used to test the cis regulatory function of thousands of DNA sequences in one experiment and can be deployed in vivo in a cell-type-specific manner [41]. The main limitation is that these ~150 bp synthetic libraries are taken out of their original context, so additional validation experiments are necessary. Looking at chromatin accessibility and MYT1L TF binding together with functional assays could provide insight into the context-dependent role of MYT1L as an activator and/or repressor.

### How does MYT1L impact progenitor proliferation?

MYT1L also seems to have mixed influences on cell proliferation in different systems. In vitro fibroblast studies have shown MYT1L overexpression suppresses cell cycle programs [11], while MYT1L KO mice displayed decreased cell proliferation in the developing cortex [15]. If MYT1L is expressed in postmitotic neurons and at very low levels in fibroblasts and progenitors, how does MYT1L regulate cell proliferation in each of these cell types? During fibroblast transdifferentiation to neurons, MYT1L’s repressive role on cell cycle programs might be the by-product of the transdifferentiation process since related pathways, like Notch signaling, are suppressed during neuronal differentiation [42]. The nature of the MYT1L-ASCL1-BRN2 fibroblast overexpression system makes it hard to delineate normal functions of MYT1L itself. Because of this, MYT1L KO mice might help better understand how MYT1L regulates cell cycle under physiological conditions. One hypothesis is that the effect is cell autonomous: that MYT1L is expressed in a small proportion of intermediate progenitors (IP; which has been observed in [15]), and this prevents early differentiation of IPs to mature neurons, thus maintaining progenitor pools. Thus, loss of MYT1L leads to precocious IP differentiation and depletion of progenitors in the developing mouse cortex (Fig. 2C). An alternative, non-cell autonomous hypothesis is that MYT1L loss from differentiating neurons (which have robust MYT1L expression normally), might decrease a lateral inhibition signal that normally prevents the differentiation of nearby neural precursors—potentially via decreased Delta like ligand (DII), known to play a role in lateral inhibition [43, 44]. Both hypotheses could explain the observed down-regulation of the cell cycle programs in bulk RNA-seq experiments. To further dissect how MYT1L impacts cell proliferation either cell autonomously or non-cell autonomously, mosaic deletions and/or single-cell sequencing on MYT1L mutant mouse cortex could be applied.

### How might MYT family members interact to promote neuronal differentiation?

**Fig. 3:**
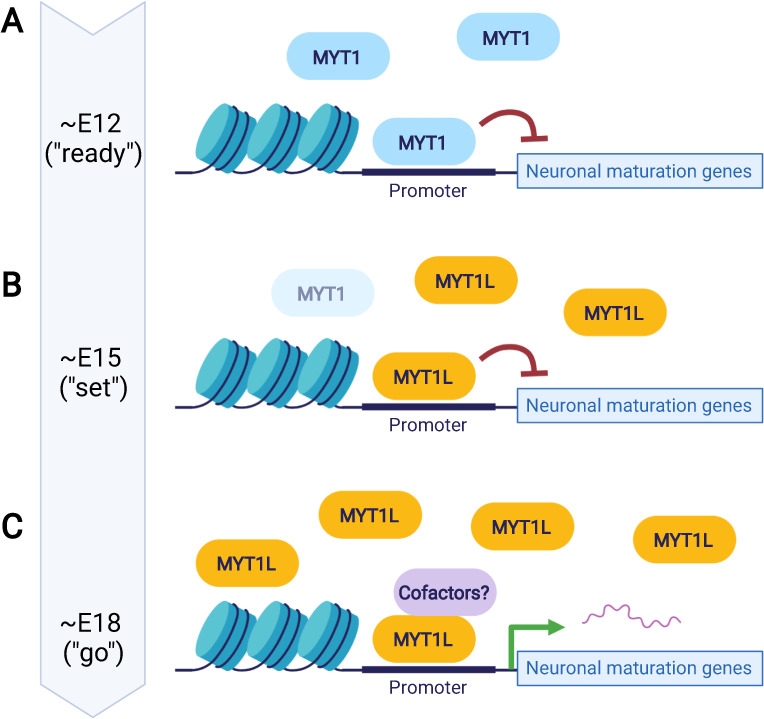
Speculative “ready-set-go” model of MYT TFs during neuronal differentiation. **A** “Ready” phase: initial expression of MYT1 during early neurodevelopment represses non-neuronal and neuronal maturation gene expression programs. **B** “Set” phase: MYT1 expression fades and is replaced by MYT1L and is still net repressive to prevent maturation gene expression. This ensures maintenance of the progenitor pool. **C** “Go” phase: MYT1L, due to possible interactions with cofactors, postransciptional modifications, or increased expression levels activates the expression of neuronal maturation genes. Figure created with BioRender.com.

How do the known expression patterns and their molecular functions as activators and/or repressors for this family of proteins inform our knowledge of their role in neurodevelopment? One model that might fit both the expression pattern, binding pattern, and what is known so far about the molecular function of the MYT family would be a “ready-set-go model” (Fig. 3). Speculatively, as progenitors get “ready” to differentiate into neurons, MYT1 may be expressed first, where it binds to and represses promoters of non-neuronal genes to silence them, and of neuronal genes to prevent them from expressing too early (i.e., so as not to form synapses during early phases of migration). As differentiation progresses (“set”), MYT1 levels fade and MYT1L begins to be expressed, so that it gradually replaces MYT1 at these same sites but remains initially repressive. However, with time, MYT1L binding at neuronal promoters transitions from repressive to activating, thus promoting (“go”) maturation in gene expression. This last transition could be mediated by the arrival of newly expressed proneuronal cofactors (e.g., NEUROD1) that could synergize with MYT1L’s N-terminal activating domain, or post-translational modification to block interaction with SIN3B, or even changes in the amount of MYT1L at a given locus over time allowing for saturation of adjacent lower affinity sites and alteration of regulatory activity. The co-factor model would allow it to become an activator at different genomic sites at different times, depending on adjacent motifs for interacting partners. While speculative, the model could explain the data so far, and is testable in future studies.

### What cell types are driving the observed clinical phenotypes?

In vitro transdifferentiation studies, in vivo knockdown experiments, and analysis of the cortex from MYT1L heterozygous mice all show that MYT1L is important for neuronal development and maturation. In general, loss or knockdown of MYT1L leads to an increase of an immature neuron transcriptional profile and decrease of a mature neuron transcriptional profile in adults. However, given the pan-neuronal expression of MYT1L, one can wonder if certain subtypes of neurons could be more sensitive to the loss of MYT1L, with distinct cell types leading to each of the diverse panel of clinical phenotypes such as obesity, white-matter thinning, hyperactivity, and social deficits. Likewise, initial results suggest that neural progenitors may be precociously differentiated, leading to a reduced pool of progenitors. However, further studies will need to be done to determine if this leads to altered final proportions of cell types in the cortex and other implicated brain regions such as the hypothalamus. In addition, the possibility of a non-cell autonomous role of MYT1L on glia, and the importance of that to patient phenotypes, remains to be explored. While the observed phenotypes during embryonic development are likely cell autonomous due to lack of mature glia at this developmental stage, further studies (e.g., single-cell sequencing) are needed to deconvolve non-cell autonomous effects.

Another possibility is that the clinical phenotypes are driven more by anomalies in the connections between cells (i.e., circuit deficits) rather than cell autonomous effects—a possibility that has so far been unexamined. Electrophysiological studies in haploinsufficient models have been limited to patch clamp of individual visual cortex neurons, which identified excitatory/inhibitory (E/I) imbalance in pyramidal neurons [15]. Thus, it would be of interest to examine circuit properties as well as functional connectivity in this system to determine if these cellular deficits result in circuit miswiring or dysfunction. Likewise, there is an interesting hypothesis from the Greenberg lab proposing the fundamental deficit in NDDs, especially for those mediated by mutations in TFs like MYT1L, is a disruption in the stereotyped pattern of activity-dependent gene expression that is required for the changes in synaptic strength underlying learning and memory [45]. An inability for gene expression to support functional changes in circuits in response to experience could also lead to anomalous behaviors in MYT1L haploinsufficient mice. Thus, there is an opportunity for better measures of circuit function and its relation to gene expression in these mutants. Such knowledge of the susceptible cell types and circuits will be key to guiding targeted strategies to rescue MYT1L function in specific cell populations for potential translational benefit.

## Concluding remarks and perspectives

Better understanding of MYT1L’s mechanism, function, and protein structure will be key for accurately interpreting how a LoF mutation can contribute to the diverse observed clinical phenotypes. As the expression of MYT1L peaks perinatally with continued low expression throughout adulthood, temporal analysis of changes arising from acute and chronic MYT1L dysfunction will be important to determine an effective therapeutic window. Finally, as MYT1L is a neuron-specific pan-neuronal TF, studying the molecular and cellular disruptions at both the single cell and circuit levels can be fruitful to identify susceptible cell populations and perturbed networks. This approach can enable focused investigations into relevant cell populations and pathogenic mechanisms, potentially resulting in the future development of targeted therapeutic strategies.

## Supplementary information

Supplemental Video 1

Supplemental Video 2
